# Supplementary material for: Integrated Analyses of m6A Regulator-Mediated Methylation Modification Patterns and Tumor Microenvironment Infiltration Characterization in Pan-Cancer
Source: Int J Mol Sci. 2022 Sep 23;23(19):11182. doi: 10.3390/ijms231911182 (PMC9570346; doi:10.3390/ijms231911182)
Supplement: Supplementary file 1 [file ijms-23-11182-s001.zip › Supplementary.pdf]

Supplementary Materials for

"Integrated analyses of m^{6}A regulator-mediated methylation modification patterns and tumor microenvironment infiltration characterization in pan-cancer"

Qingkang Cao[1], Yuanyuan Chen [1,*]

1 Department of Mathematics, College of Science, Nanjing Agricultural University, Nanjing210095, China.

* Correspondence: chenyuanyuan@njau.edu.cn

<u>**Overview of Supplementary Materials:**</u>

Supplementary Figures

Supplementary Figure S1: Kaplan-Meier curves of patients in the low-RBM15 group and the high-RBM15 group in pan-cancer.

Supplementary Figure S2: Unsupervised clustering based on 20 m^{6}A regulators. The consensus fraction matrix of all samples when k=2.

Supplementary Figure S3: Functional annotation for m^{6}A phenotype-related using KEGG enrichment analysis.

Supplementary Figure S4: Unsupervised clustering based on 37 m^{6}A phenotype-related genes. The consensus fraction matrix of all samples when k=2.

Supplementary Tables

Supplementary Table S1: Spearman correlation analysis of the 20 m^{6}A modification regulators.

Supplementary Table S2: Samples clustering in pan-cancer.

Supplementary Table S3: Enrichment score of KEGG pathways in pan-cancer.

Supplementary Table S4: TME infiltration characteristics of samples in pan-cancer.

Supplementary Table S5: The correlation of m^{6}A modification regulators and TME infiltration Characteristics.

Supplementary Table S6: The Univariate Cox regression analysis of 40 differential genes.

Supplementary Table S7: Functional annotation for m^{6}A phenotype-related genes.

Supplementary Table S8: Gene signatures used in this study for marking biological processes.

Supplementary Table S9: The gene sets used in this work for marking 28 types immune infiltration cells.

Supplementary Figures

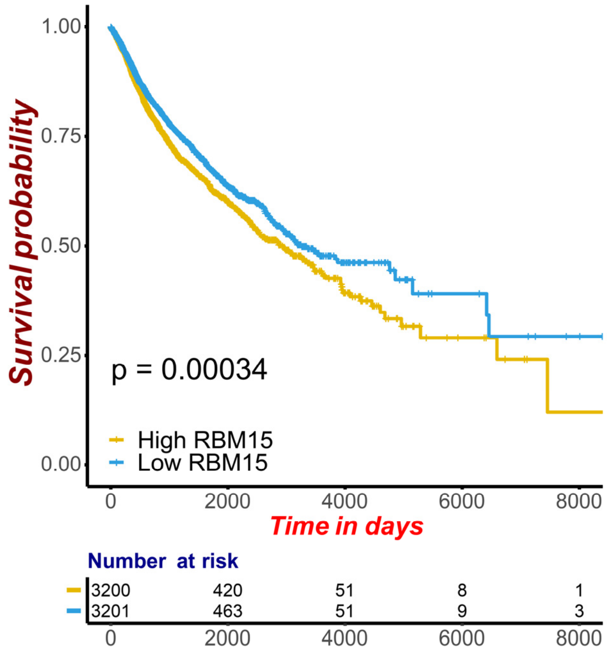

Figure S1. Kaplan-Meier curves of patients in the low-RBM15 group and the high- RBM15 group in pan-cancer. The X-axis shows survival time in days. The Y-axis shows the overall survival rate.

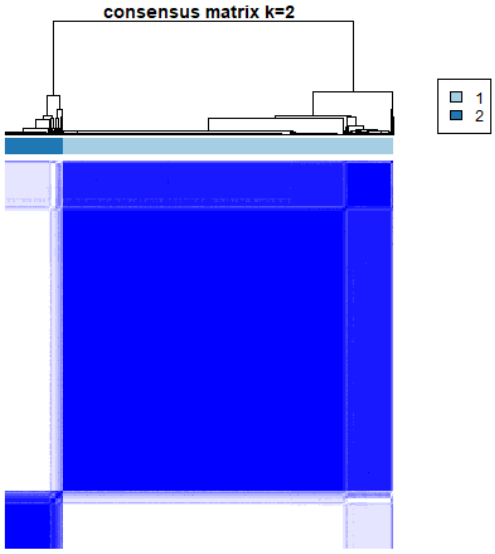

Figure S2. Unsupervised clustering based on 20 m^{6}A regulators. The consensus fraction matrix of all samples when k=2.

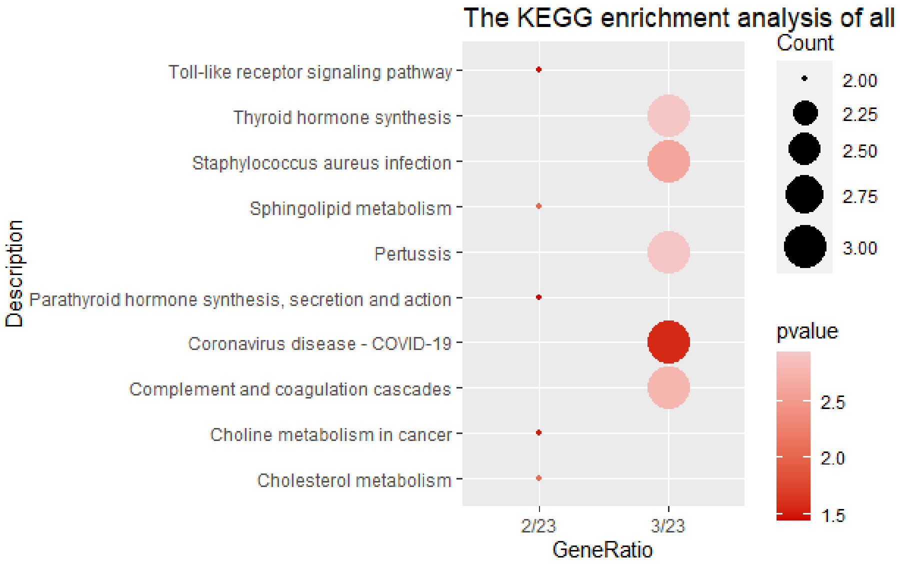

Figure S3. Functional annotation for m^{6}A phenotype-related genes using KEGG enrichment analysis.

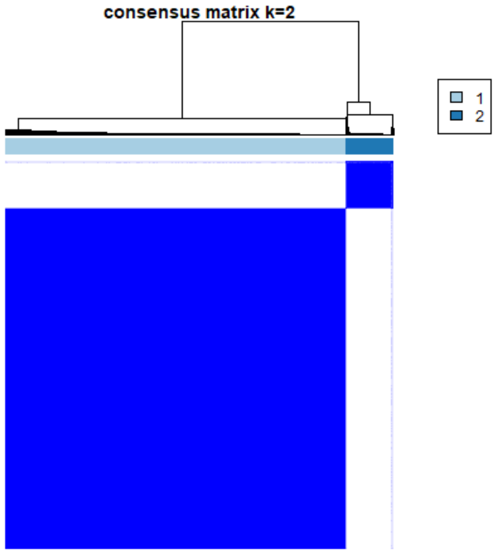

Figure S4. Unsupervised clustering based on 37 m^{6}A phenotype-related genes. The consensus fraction matrix of all samples when k=2.
